# Improving the accuracy and efficacy of diagnosing polycystic ovary syndrome by integrating metabolomics with clinical characteristics: study protocol for a randomized controlled trial

**DOI:** 10.1186/s13063-020-4060-6

**Published:** 2020-02-11

**Authors:** Cheng-Ming Ni, Wen-Long Huang, Yan-Min Jiang, Juan Xu, Ru Duan, Yun-Long Zhu, Xu-Ping Zhu, Xue-Mei Fan, Guo-An Luo, Yi-Ming Wang, Yan-Yu Li, Qing He, Lan Xu

**Affiliations:** 1 Department of Endocrinology, The Affiliated Wuxi People’s Hospital of Nanjing Medical University, Nanjing Medical University, Wuxi, 214023 China; 2 Department of Good Clinical Practice (GCP), The Affiliated Wuxi People’s Hospital of Nanjing Medical University, Wuxi, 214023 China; 3 Department of Endocrinology, Jiangyin People’s Hospital, Wuxi, 214400 China; 4 Department of Endocrinology, The Affiliated Wuxi Maternal and Child Health Centers Clinical Hospital of Nanjing Medical University, Wuxi, 214023 China; 5 Key Laboratory of Bioorganic Phosphorus Chemistry and Chemical Biology (Ministry of Education), Department of Chemistry, Tsinghua University, Beijing, 100000 China

**Keywords:** Polycystic ovary syndrome, Metabolomics, Clinical signs and symptoms, Accuracy and efficacy

## Abstract

**Background:**

Polycystic ovary syndrome (PCOS) is a complex endocrine syndrome with poorly understood mechanisms. To provide patients with PCOS with individualized therapy, it is critical to precisely diagnose the phenotypes of the disease. However, the criteria for diagnosing the different phenotypes are mostly based on symptoms, physical examination and laboratory results. This study aims to compare the accuracy and efficacy of diagnosing PCOS by integrating metabolomic markers with common clinical characteristics.

**Methods:**

This is a prospective, multicenter, analyst-blinded, randomized controlled trial. Participants will be grouped into (1) people without PCOS (healthy control group), (2) patients diagnosed with PCOS based on clinical indices (experimental group 1), and (3) patients diagnosed with PCOS based on metabolomic indices (experimental group 2). A total of 276 participants, including 60 healthy people and 216 patients with PCOS, will be recruited. The 216 patients with PCOS will be randomly assigned to the two experimental groups in a 1:1 ratio, and each group will receive a different 6-month treatment. The primary outcome for the experimental groups will be the effect of PCOS treatment.

**Discussion:**

The results of this trial should help to determine whether using metabolomic indices is more accurate and effective than using clinical characteristics in diagnosing the phenotypes of PCOS. The results could provide a solid foundation for the accurate diagnosis of different PCOS subgroups and for future research on individualized PCOS therapy.

**Trial registration:**

Chinese Clinical Trial Registry, ID: ChiCTR-INR-1800016346. Registered 26 May 2018.

## Background

Polycystic ovary syndrome (PCOS) is a complex endocrine syndrome [1, 2] that exhibits chronic ovulatory disorders, hyperandrogenism, insulin resistance, and metabolic disorders [3]. PCOS has a prevalence of 5%–10% globally [4, 5], and it is one of the most common endocrine and metabolic disorders in Chinese women. PCOS is strongly associated with insulin resistance and dyslipidemia, which affect patients with obesity, type 2 diabetes, and metabolic syndrome (MetS) [6–10]. Evidence also suggests that patients with PCOS may have a higher prevalence of asthma [11], nonalcoholic fatty liver disease [12], and mental disorders [13] such as depression and anxiety [14]. However, the underlying mechanisms of PCOS are still poorly understood, making clinical diagnosis and treatment very difficult.

To determine whether a patient has PCOS, careful assessment of hyperandrogenism, hyperandrogenemia, ovulatory function, and ovarian morphology is required. The recent diagnostic criteria for PCOS are mostly based on a combination of other criteria/statements that are still disputed, and because of the different clinical manifestations and complex pathogenesis of the disease, the criteria are mainly aimed at ruling out other diseases. The accuracy of the diagnosis and the evaluation methods for assessing the individual criteria is even more critical.

PCOS has diverse phenotypes [15–17] and thus requires individualized treatment. The European Society of Endocrinology [2] proposed four phenotypes, including a type without hyperandrogenemia. When diagnosing the disease, it was suggested that abnormalities in steroidogenic enzymes, prolactin excess, and thyroid problems need to be ruled out. In China, the classification of subtypes and treatment of PCOS are mainly based on clinical indices, such as sex hormones (e.g., follicle-stimulating hormone (FSH), luteinizing hormone (LH), LH/FSH ratio, free testosterone, dehydroepiandrosterone (DHEAS), androstenedione, sex-hormone-binding globulin), serum lipids (e.g., total cholesterol, triglycerides, low-density lipoprotein cholesterol (LDL-C), high-density lipoprotein cholesterol (HDL-C), apolipoprotein (Apo) A, Apo B), and the results of oral glucose tolerance tests and immunoreactive trypsinogen tests [1]. This heterogeneity in diagnostic criteria could significantly lead to misdiagnoses. Therefore, it is urgent to explore a new detection method to standardize the classification of phenotypes and establish an efficient diagnostic and treatment strategy for PCOS. The development and application of new technologies, such as genomics, proteomics, and metabolomics, provide a new direction for diagnosing PCOS phenotypes. However, in genomics, although the findings of genome-wide association studies have implications, the direct pathogenic gene for PCOS has not been found yet. In proteomics, some scholars have identified potential biomarkers for PCOS phenotyping, but their clinical application is limited because proteins, or amino acids, are highly susceptible to interference, which can bias the results. Lastly, due to the high sensitivity and specificity of metabolomic methods, it can be assumed that some metabolic markers can be used to distinguish whether patients with PCOS have high androgen levels.

In the attempt to classify PCOS phenotypes, recent studies have adopted various measurements such as nuclear magnetic resonance [18], liquid chromatography/mass spectrometry [19], and others [20, 21]. In our previous study [3], using ultra-high-performance liquid chromatography/quadrupole time-of-flight mass spectrometry, we found two subgroups of patients with PCOS: one with hormone metabolism disorders and the other with lipid metabolism disorders.

This trial aims to compare the accuracy and efficacy between using metabolomic indices and using clinical characteristics in diagnosing PCOS phenotypes. Our findings could provide a basis for further research on individualized PCOS therapy.

## Methods

### Study design

This is a prospective, multicenter, analyst-blinded, randomized controlled trial. Subjects will be grouped into one healthy control group and two parallel experiment arms, as follows: (1) people without PCOS (healthy control group), (2) patients diagnosed with PCOS based on clinical indices (experimental group 1), and (3) patients diagnosed with PCOS based on metabolomic indices (experimental group 2). A total of 276 participants, including 60 people without PCOS and 216 patients with PCOS, will be recruited. The 216 patients with PCOS will be randomly assigned to the two experimental (diagnosis) groups in a 1:1 ratio. After group assignment, medical detection results, relative information, and biological samples will be collected from all three groups. Additionally, the blood samples from all participants will be tested using metabolomic methods for further study, while the analysis results will be totally blinded to all participants and their research physicians. Based on our previous study [3], we will categorize patients with PCOS into two subgroups: patients with hormone metabolism disorders (subgroup 1) and patients with lipid metabolism disorders (subgroup 2).

The two diagnosis groups will be automatically divided into the two different subgroups according to patient characteristics. The main difference is that in group 1, diagnosis will be based on clinical indices [1], such as clinical signs and symptoms and ultrasonography findings, while in group 2, diagnosis will be based on metabolomic indicators. All patients will receive treatment according to their subtype group, and the treatment will consist of Diane-35 or metformin. The intervention period for all experimental groups will be 6 months, and the results will be evaluated after the completion of three constant treatment sessions (3 months).

**Fig. 1:**
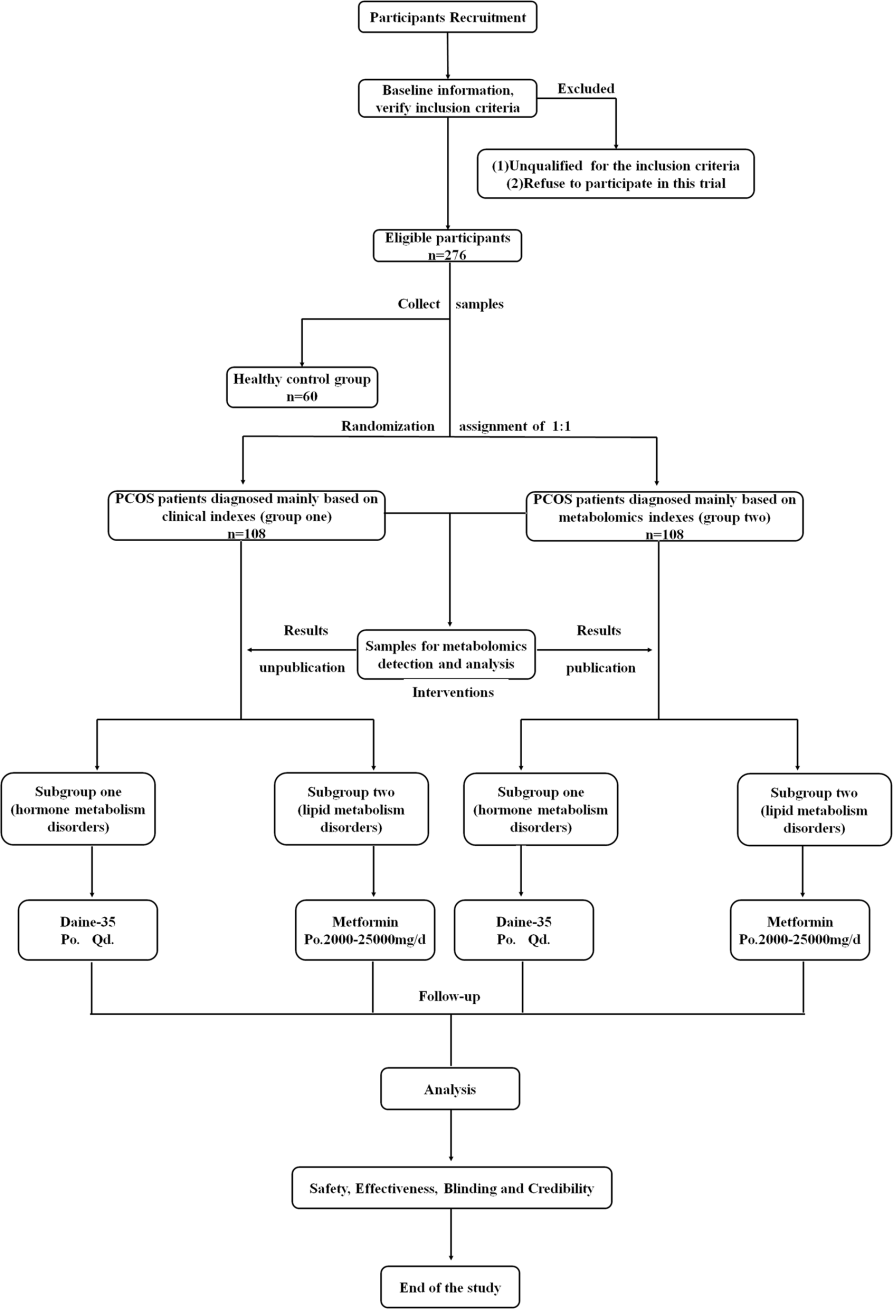
Flow chart diagram showing the summary of the trial design

**Fig. 2:**
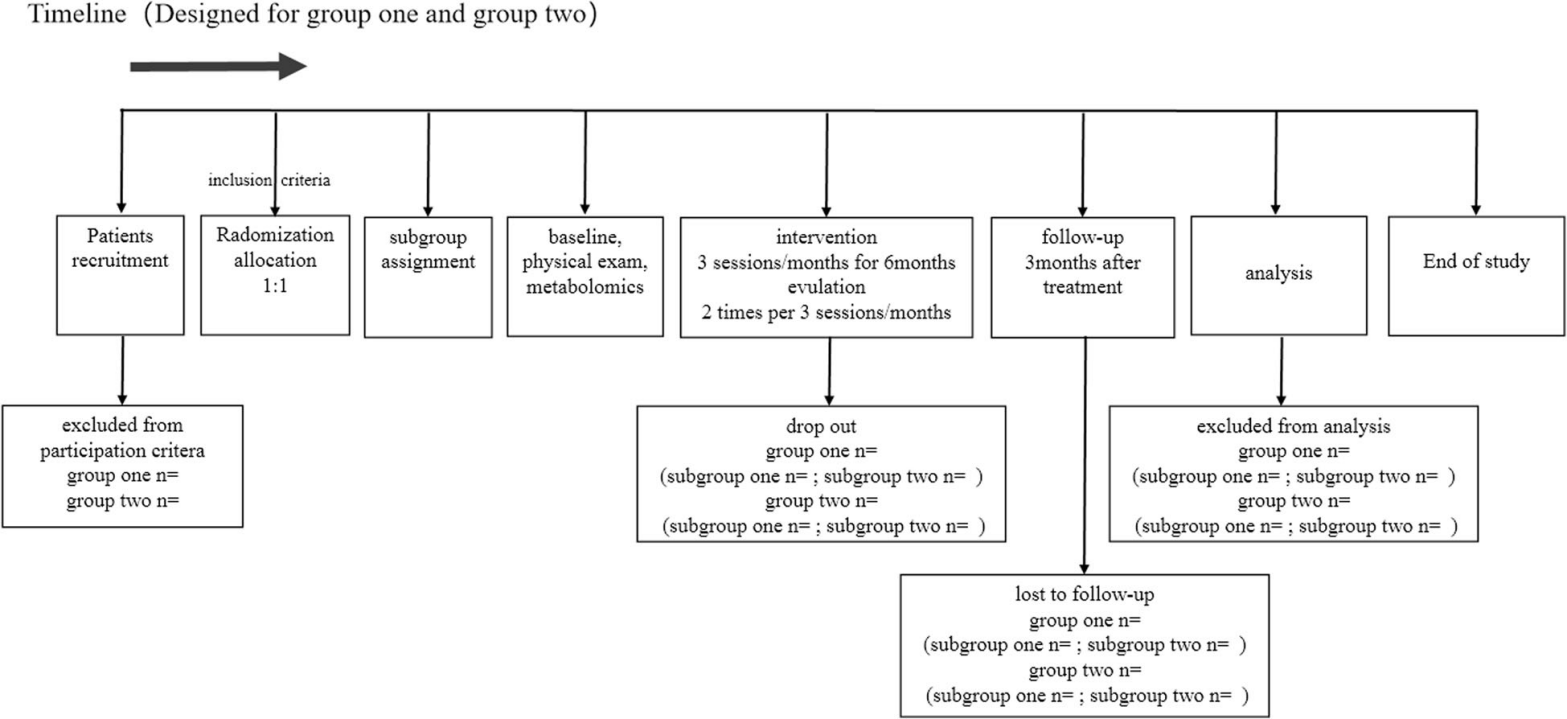
Timeline of the the trial

**Fig. 3:**
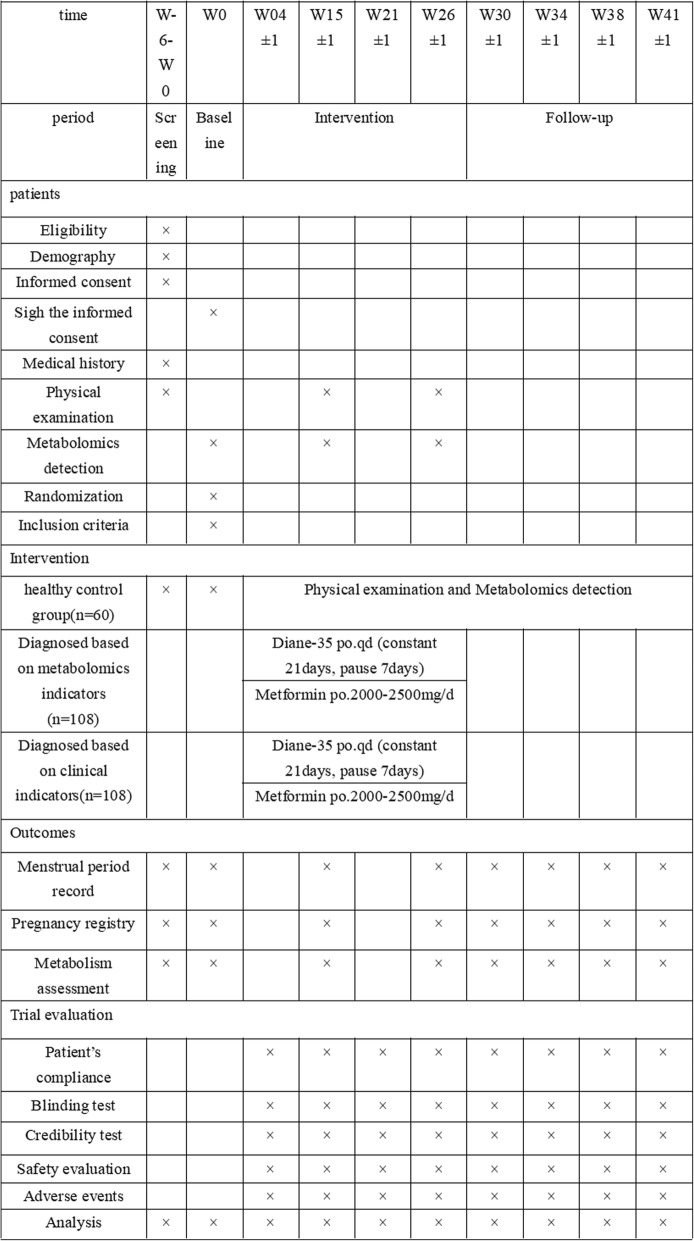
The schedule of forms and procedures, according to the Protocol Items. Physical examination includes: ultrasonography of bilateral ovarian anad clinical biochemical indicators. Clinical biochemical indicators routine urine tests, routine blood tests, liver function, pregnancy tests, LH/FSH ratio, sex hormone related indicators blood lipid related indicators and endocrine related indicators. There are twenty-five clinical biochemical indexes including eight sex hormone-related indexes: FSH, LH, PRL, E2, P, Ts, AMH, AND vitD; nine blood lipid indicators: TC, TG, HDL, LDL, ApoA1, Apo B, Lp(a), FFA, hsCRP; seven endocrine-related indicators: GLU (0h, 30min, 120min), INS (0h, 30min, 120min), BUA. The main elevation index was LH/FSh ratio. Note: ultrasonography examination is scheduled for the 14th day of menstruation, if the patient with rare ovulation has follicular diameter > 10mn or luteal appearance, should be reviewed in the subsequent menstrual cycle. Unmarried women without sexual life suggested abdominal B ultra. All blood samples will be collected on an empty stomach within 72 hours from the first day of menstruation

Outcomes will be assessed, and the data will be analyzed by someone blinded to the assignment of participants. The study design is shown in Fig. 1, and the timeline is presented in Figs. 2 and 3.

The main treatment center will be the Department of Endocrinology and Metabolism of the Affiliated Wuxi People’s Hospital of Nanjing Medical University, which will enroll 60 participants for the healthy control group and 108 participants who, in the experimental group (108/216), will be randomly assigned between the two experimental groups. Two other treatments centers—Wuxi Maternal and Child Health Centers Clinical Hospital and Jiangyin People’s Hospital—will enroll the other 108 participants, who will also be randomly assigned between the two experimental groups.

### Participants and recruitment

People without PCOS will be enrolled in the healthy control group only if they do not have any other disorders that may influence sex-related hormones and metabolic conditions. For the experimental groups, PCOS will be diagnosed according to the 2018 Chinese Endocrine Society clinical practice consensus on PCOS [1], which is based on the widely accepted criteria for PCOS published by Rotterdam [22, 23]. Patients who are of reproductive age and can follow the procedure, including strict contraception for 6 months, will be informed of this trial. If a potential participant expresses interest, a face-to-face interview regarding the entire trial process will be conducted in a reception room in the three hospitals. Patients who meet the inclusion criteria will be enrolled if they provide written informed consent. Potential participants will be recruited in two ways: (1) the experimental group participants will be recruited by approaching patients with PCOS who are admitted to an outpatient department or inpatient ward of each center; the official microblog and WeChat platforms of each center will also be used; and (2) the healthy control group participants will be directly recruited by approaching volunteers in the health examination center of Wuxi People’s Hospital.

### Inclusion criteria

Participants who meet all of the following criteria will be enrolled in the experimental groups:

1. Diagnosed with PCOS according to the 2018 Chinese Endocrine Society clinical practice consensus, i.e., presence of at least two of the following three conditions, excluding other etiologies: (a) clinical or biochemical hyperandrogenism, (b) oligo-anovulation (menstrual cycle length of > 35 days and having eight or fewer menstrual cycles per year), and (c) a polycystic ovarian morphology characterized by at least 25 small follicles (2–9 mm) in the entire ovary and/or an increase in ovarian volume of ≥ 10 mL
2. A reproductive woman aged 18–45 years
3. Able and willing to comply with the interventions and follow-up evaluations

### Exclusion criteria

The exclusion criteria are as follows:

1. Currently receiving treatment in another experimental study or having just finished a trial in the past 30 days
2. Treated with Diane-35, metformin, or other forms of estrogen, progesterone, and lipid-regulating and hypoglycemic drugs (e.g., glucocorticoids, spironolactone, antibiotics, bacterial regulators, anti-inflammatory drugs) within the past 12 weeks
3. An allergy or intolerance to Diane-35, metformin, or any of its components
4. Presence of congenital adrenal cortical hyperplasia, hypercorticism, androgen-secreting tumors, Cushing’s syndrome, thyroid dysfunction, gonadotropin deficiency, hyperprolactinemia, premature ovarian insufficiency, functional hypothalamic amenorrhea, or diabetes
5. A medical history of malignant tumors, particularly a history of surgery, radiation, or chemotherapy for a gynecological malignant tumor
6. Presence of liver dysfunction (alanine aminotransferase and aspartate transaminase levels > 1.5-times the normal values) or chronic liver disease
7. Presence of hematopenia or thrombotic disease
8. Is pregnant or expects to be pregnant during the study
9. Has an unstable medical, physical, or mental status
10. Has a medical history of symptomatic ventricular arrhythmias with torsion ventricular tachycardia (torsade de points)
11. Unable to complete the procedures of the study
12. Any other situation that would interfere with the study evaluation, procedures, or completion

### Subject dropout

Subject dropout occurs when:

1. The subject quits the study
2. Safety issues arise (e.g., adverse events (AEs), failure of contraceptive measures, accidents)
3. The subject is lost to follow-up
4. Researchers remove the subject from the trial due to poor compliance, severe liver dysfunction, complications, or serious AEs

### Suspension of the trial

The trial may be suspended if the following events occur:

1. A significant safety problem is found, such as serious impairment of the patients’ liver function or the sudden onset of other life-threatening illnesses. In this case, the trial will be suspended, and the participants will have to undergo medical treatment until recovery
2. The diagnostic and therapeutic effects are poor, such as no improvement in menstrual/metabolic disorders (to be evaluated at the third month of therapy). In this case, the researchers will evaluate the physical condition of the participants and decide whether to suspend the trial if necessary
3. A major mistake (e.g., patients taking the wrong doses, patients taking or using medicine without following the physician’s advice) is made that will affect the results of the trial
4. There is a major problem in funding or management, such as withdrawal of funding, which can cause the trial to be discontinued early

### Randomization

The Department of Good Clinical Practice of Wuxi People’s Hospital will lead the randomization process by using a random number generator in the Statistical Package for Social Sciences (SPSS) version 21.0 (SPSS Inc., Chicago, IL, USA).

Random sequences will be placed in opaque envelopes, numbered in order, and then sent to a clinical researcher and physicians.

The envelopes will be opened sequentially to decide upon the allocation of participants. In this trial, the physicians and trial participants will be blinded to the group assignment.

### Blinding

In the experimental groups, participants will not be informed of the group assignment, the type of diagnosis, or the treatment that they will receive. Data managers and the statistician will also be blinded. Only the trial administrator (a steering committee of the Department of Good Clinical Practice, Wuxi People’s Hospital), who will be responsible for assigning the participants into different subgroups and monitoring the entire trial process, will be not blinded. Moreover, the clinical research physicians will be blinded to the assignment of group 1, whose treatment will be decided based on their clinical practice, while they will have access to the diagnostic and treatment allocation data for group 2. The clinical physicians will be responsible for learning how to use the blinding method to communicate with the participants to ensure diagnosis and treatment blinding. The blinding procedure will be conducted until the data are locked and the trial is completed. Unblinding will only be permitted in case of a medical emergency or when a participant quits the trial. All cases of unblinding will be documented.

### Interventions

#### Healthy control group

As mentioned above, participants will only be recruited in the healthy control group if they do not have PCOS, metabolic conditions, or any other disorders that may influence sex-related hormones and they are not using any drugs that could also influence sex hormones. A medical evaluation will be conducted to confirm eligibility, after which the trial team will collect information regarding the patient’s medical history, conduct a physical examination, and collect blood samples from eligible participants for further investigation.

#### Clinical index group

For the clinical index group, blood samples and medical information of all eligible participants will be collected. Based on several guidelines and clinical experience, subgroup 1 (participants with hormone metabolism disorders) will receive orally administered Diane-35 once a day for 21 days, after which the treatment will be paused for 7 days. Subgroup 2 (participants with lipid metabolism disorders) will receive orally administered metformin (2000–2500 mg) once a day based on their clinical condition. The entire treatment period will be at least 6 months. During the trial, blood samples will be collected three times (week 0, week 15 ± 1, and week 26 ± 1) for metabolomic evaluation to assess changes. However, the detection information will be blinded to all participants and clinical research physicians to avoid influencing the results. All clinical research physicians will be required to have majored in endocrinology and metabolism or gynecological endocrinology for at least 10 years. They must also be employed as an attending physician for > 5 years. Finally, they must receive professional training on clinical trials and pass a test to ensure consistent performance in the study methods.

#### Metabolomic index group

Most of the interventions for the metabolomic index group will be similar to those for the clinical index group. The main difference is that the division of the subgroups will be based on metabolomic indices. The physicians in this group will be informed of the allocation of participants to the subgroups and will provide the corresponding treatment scheme to all participants. The physicians will not be allowed to change the scheme during the trial and will remain blinded to the metabolomic results to minimize the possibility of any influence on treatment decisions.

### Concurrent treatment of patients

All other treatments for PCOS will be banned during the trial, including oral contraceptives, broad-spectrum lipid drugs, and any other drugs that might influence the results. However, participants may receive any treatment not related to PCOS and will be instructed to use condoms during sexual intercourse. Any change in concurrent treatment will be recorded at every visit.

### Outcome measurements

The healthy control group will remain enrolled in the trial until all their samples are collected. For the experimental groups, the primary outcome will be the changes in their relative PCOS condition evaluated using clinical indices. The main clinical index that will be evaluated is the LH/FSH ratio.

Secondary outcomes will be assessed using other indices, signs, and symptoms, as described in the 2018 Chinese Endocrine Society clinical practice consensus on PCOS [1] such as sex hormone-related indices (FSH, LH, prolactin, estradiol, progesterone, testosterone, anti-Müllerian hormone (AMH), and vitamin D), blood lipid-related indicators (total cholesterol, triglycerides, HDL, LDL, Apo A1, Apo B, lipoprotein(a), free fatty acids, and high-sensitivity C-reactive protein), and endocrine-related indicators (glucose and insulin (at 0, 30, and 120 min) and blood uric acid). To help evaluate the outcomes, three metabolic markers will also be used, namely, palmitoyl sphingomyelin, cyclic guanosine monophosphate, and DHEAS. Ultrasound results will also be evaluated, and the number and diameter of follicles in both ovaries will be measured.

Safety outcomes will include any severe impairment in liver function or inability of the patient to tolerate symptoms (e.g., nausea and vomiting caused by metformin) related to the therapeutic drugs. Such outcomes will be monitored after each treatment session (1 month) using the hepatic function test. An exploratory outcome will be complete recovery from PCOS after treatment within 26 weeks.

### Safety evaluation

AEs will be evaluated at every visit and will include any unexpected or unfavorable response that occurs during or after treatment. Although these events may not have a causal relationship with the variables in this study, the investigators will need to ensure that all AEs are reported and recorded in the subject’s medical records. In this trial, AEs are defined as disorders that (1) hinder one’s ability to work or are life-threatening (especially liver dysfunction) or (2) lead to hospitalization or prolong department or hospital stay. Remedial treatment should be given immediately to resolve any observed AE, and all AEs will be reported to the responsible units, ethical committees, and trial administrator to decide whether the participant ought to remain in or drop out of the trial. Regardless of the decision, all participants with an AE will be followed up until the event has been resolved or the condition has become chronic or stable.

The study should not cause any additional harm to the participants. In case of serious unexpected harm to a participant related to the study, we will shoulder the relevant expenses (including the diagnosis and treatment) and follow up the condition of the participant until recovery.

### Follow-up

To evaluate the accuracy and efficacy of diagnosis, safety, and the superior effects of individual interventions, a follow-up via telephone (or via face-to-face interviews, emails, text messages, or WeChat) will be conducted 6 months after the trial. During the follow-up, no participants will undergo special therapy except for routine cervical care. At weeks 30, 34, 38, and 41, the outcome assessor will call the participants to investigate their PCOS condition, asking mainly about menstrual cycle length and frequency, the appearance of hyperandrogenism (e.g., weight loss, acne, excessive facial and body hair), and pregnancy. Participants are welcome to inform the assessors of their clinical symptoms and AEs via face-to-face meetings, emails, telephone calls, text messages, or WeChat at relevant time points.

### Blinding and credibility tests

Metabolomic evaluation with blinding will be completed at weeks 0, 15, and 26. The implementation of the blinding strategy will be crucial for the trial. The credibility rating for both the diagnostic methods and different types of treatment will be estimated using a credibility test at weeks 15, 26, and 41 during the follow-up period.

### Data collection and monitoring

Data on basic patient characteristics will be collected and recorded by screeners when participants are recruited. Clinical signs and symptoms, physical examination findings, metabolomic evaluation results, short- and long-term outcomes, assessment of diagnosis accuracy and treatment efficiency, and details of the AEs will be recorded by trial assessors and clinical researchers in case report forms (CRFs).

Completed CRFs will be checked and reviewed by a five-person steering committee, which is composed of two supervisors of the major trial center, a data statistician, and two data administrators. The committees are completely independent from the research team and will be constantly blinded to the group allocation.

All data entry and management procedures will be conducted in an OpenClinica System (version 3.12) database. The committee members will need to be qualified in data analysis and will have been trained uniformly.

To ensure the accuracy of data, two data administrators will independently perform data entry and validation. Any issues with the information in the CRFs will be reported by the data administrators to the steering committee. Any revisions will be modified by the administrators according to feedback from the committee. Once the accuracy of data is confirmed, the electronic database will be locked while real-time tracking and monitoring will remain open.

The steering committee and the Department of Good Clinical Practice in Wuxi People’s Hospital will monitor the entire trial (e.g., checking the progress of recruitment, participant data including CRFs, the protocols for researchers, informed consent forms, and any other study-specific files) and audit the trial conduct at least once a month.

All information will be made available to the investigators whose proposed use of the data has been approved by the relevant committee for up to 15 years following publication. The findings will be presented at conferences and published in peer-reviewed journals. A summary of the findings will be provided to the participants upon request via telephone.

### Sample collection and handling

The actual dates and times of sample collection will be recorded in the laboratory requisition form. Instructions for the collection, handling, and storage of samples are found in the laboratory manual that will be provided.

The standard procedure for plasma separation will be performed as follows:

1. Fresh anticoagulated peripheral blood will be collected in the EDTA anticoagulant tube
2. The fresh peripheral blood will be centrifuged at 1700 G at 4 °C for 10 min, and the upper plasma will be carefully collected to avoid touching the lower layers of red blood cells and white blood cells
3. The collected plasma will be centrifuged at 2000 G at 4 °C for 10 min, and the supernatant will be decanted
4. The plasma will be divided and placed in separate containers and frozen at − 80 °C in a refrigerator for later use

### Precautions for plasma separation

1. The plasma separation process shall be performed on ice to maintain the integrity of the blood cells
2. The entire separation process will be completed within 4 h
3. The blood collection and plasma separation process will be handled carefully to avoid the risk of hemolysis

After the study, all samples will be safely destroyed.

### Statistical analyses

The dataset will include a safety set, a full analysis set (FAS), and a per-protocol set. The safety set is designed for participants who will be randomly assigned to receive at least one session of treatment. The FAS will include all medical data related to the trial and will indicate the intervention conducted; however, participants who miss the primary outcome evaluation will be excluded. In the per-protocol set, participants will be included if they receive at least three constant sessions of treatment.

In this trial, the FAS will be used for the basic analysis. For missing data, we will employ the imputation adjustment approach, and the last observation analysis will be selectively used to handle the missing data.

A sensitivity analysis will then be used to compare the results of the per-protocol analysis and the different intervention analyses to evaluate the impact of the missing data on the trial results.

Descriptive statistics about quantitative indicators, such as means, standard deviations, medians, minimums, and maximums, will be presented. Variance analysis will be used to compare the quantitative indices among the different groups (healthy control group and experimental groups 1 and 2), and the Student-Newman-Keuls test will be used to analyze the two subgroups.

Classification indicators will be described by the number and percentage of each category. The chi-squared test or exact probability method will be used to compare the qualitative indices among the three groups, and the chi-squared segmentation method will be used to compare the two pairwise subgroups.

SPSS version 21.0 will be used to analyze all trial data. All statistical tests will be double-sided, and statistical significance will be considered when the *p* value is ≤ 0.05.

### Sample size calculation

Based on the literature and findings from a small pilot study that used metabolomic techniques, the sensitivity and specificity required to classify the control and PCOS groups will be 100% and 86%, respectively. The corresponding values between the control group and subgroup 1 will be 96.7% and 100%, respectively, while those between the control group and subgroup 2 will be 100% and 86.2%, respectively. The sensitivity and specificity for dividing PCOS into two different subtypes will be 90.9% and 87.5%, respectively. To meet the lower statistical limit, each subgroup will include at least 30 participants.

According to a presumptive maximum dropout tolerance of 20%, with a significance level of 0.05 and a power of 0.80, we calculate the required sample size for the experimental groups to be 216. In this trial, 60 people will be assigned to the healthy control group and 108 people to each experimental group across the three centers.

### Quality control

During the trial, quality control will be carried out by the steering committee. All researchers are required to attend training on trial methods, techniques, and protocol regulations to maintain data consistency and validity. Any modifications or corrections required will be discussed, decided upon, and submitted to the steering and ethics committees. Patient details will be kept confidential throughout the trial.

## Discussion

PCOS is a syndrome characterized by various signs and symptoms, such as androgen excess, ovarian dysfunction, and a polycystic ovarian morphology. Considering its high prevalence among reproductive women in China and given that its complications may lead to endometriosis, endometrial cancer, or some malignant diseases, PCOS might be one of the most common and severe endocrine and metabolic disorders.

The diagnosis of PCOS still depends on ruling out other diseases [24–29] including thyroid dysfunction, nonclassical congenital adrenal hyperplasia, and androgen-secreting tumors. PCOS has several recognized phenotypes that present with distinct characteristics and require individualized treatment [1]. However, with the lack of classification criteria, it is very difficult for physicians to make a precise diagnosis and develop a suitable treatment strategy.

Of note, researchers have attempted to determine whether there are biomarkers that could be used as predictive markers for the different PCOS phenotypes. Zhang et al. [30] found that the phenotype with hyperandrogenism is associated with a higher score in the homeostatic model assessment of insulin resistance (HOMA-IR) than the other phenotypes (without androgen excess). Further detection showed that serum irisin levels are associated with hyperandrogenism but not with oligo-anovulation or PCOS morphology. Additionally, Minooee et al. [31] pointed out that the testosterone-to-androstenedione ratio is associated with insulin resistance (sensitivity 0.83, specificity 0.42) and MetS (sensitivity 0.85, specificity 0.70) among patients with PCOS, indicating the strong link between insulin resistance and hyperandrogenism. This finding may assist clinicians and researchers in classifying the specific phenotypes of PCOS. However, Bozic-Antic et al. [32] designed a cross-sectional clinical study of 365 women with PCOS and 125 healthy controls from a Caucasian population. In their study, patients with PCOS were divided into groups based on four phenotypes, according to the criteria of the European Society of Human Reproduction and Embryology (ESHRE)/American Society for Reproductive Medicine (ASRM) [33]. Their findings suggested that all phenotypes are associated with the same prevalence of MetS, but phenotype B was associated with a different cardiovascular risk. This difference might partly be related to epigenetic and environmental influences. Moreover, the free androgen index [34], serum AMH level [35–38], PPARGC1A (a leukocyte methylation promoter) [39], and some other indices were also found to be potential biomarkers for predicting PCOS and classifying its phenotypes. Although there have been many efforts to address the predicament surrounding the diagnosis of PCOS, the precise classification criteria have yet to be developed. However, there has been a turning point in the detection methodology with the advent of metabolomics.

This trial is designed to explore a more specific and sensitive method to determine the subtypes of PCOS. Based on the preliminary study, our team found that clinical indicators such as the LH/FSH ratio and Apo A1, AMH, and insulin levels had a good correlation with metabolomic results. This finding indicates the potential of developing a diagnostic and therapeutic strategy for PCOS based on an integrated biomarker system.

To further investigate the findings of this trial, we will conduct a prospective cohort study, expanding the sample size of patients with PCOS and analyzing the detection frequencies. This study is a multicenter, randomized, open-label, blinded clinical study aiming to standardize the diagnostic classification and treatment of PCOS. The sensitivity and specificity of integrating metabolomic indices in the diagnostic criteria will be compared with diagnosing the disease with clinical indices alone. Our findings may help improve the empirical classification, find predictive biomarkers, and assist in establishing an integrated biomarker system for developing precise diagnostic and treatment strategies for different PCOS subgroups in the future.

### Study limitations

The main difficulty inherent to this trial is making a consistent diagnosis in experimental group 1 because the diagnosis will be made by several different physicians. To address this problem as much as possible, our group will organize a diagnosis team consisting of three professional physicians who have majored in endocrinology and metabolism or gynecological endocrinology for at least 10 years and achieved the position of assistant director . If the clinical research physicians cannot diagnose the participant’s subtype, the case will be submitted to the team for a final diagnosis. Another methodological difficulty is ensuring that the treatment complies with the scheme. In this trial, we will ensure that medication reminders are sent and participants who miss treatment for seven consecutive days will be excluded from the trial.

## Trial status

This trial will use protocol version 2.0. Recruitment was initiated in February 2019 and completed in August 2019. The trial is currently in the screening phase, and some participants are undergoing observation. Ethical approval was granted in 2018, and the study is expected to be completed at the end of December 2019.

## Data Availability

The datasets (including the metadata and protocol) analyzed in this study will be available from the corresponding author upon reasonable request and through a public clinical trial management platform (http://www.medresman.org). Sponsor members of the research team, namely Qing He and Lan Xu, and core members, namely Cheng-Ming Ni and Xue-Mei Fan, are eligible to write papers relevant to this trial. We do not intend to use any professional writers.

## Abbreviations

AE: Adverse event
AMH: Anti-Müllerian hormone
CRF: Case report form
DHEAS: Dehydroepiandrosterone
FAS: Full analysis set
FSH: Follicle-stimulating hormone
LH: Luteinizing hormone
MetS: Metabolic syndrome
PCOS: Polycystic ovary syndrome
SPSS: Statistical Package for Social Sciences
